# Low Interferon Relative-Response to Cytomegalovirus Is Associated with Low Likelihood of Intrauterine Transmission of the Virus

**DOI:** 10.1371/journal.pone.0147883

**Published:** 2016-02-16

**Authors:** Yifat Eldar-Yedidia, Maskit Bar-Meir, Miriam Hillel, Guila Abitbol, Eti Broide, Roni Falk, Marc Assous, Yechiel Schlesinger

**Affiliations:** 1 Research Laboratory of Infectious Diseases, Shaare Zedek Medical Center, affiliated to the Hebrew University Medical School, Jerusalem, Israel; 2 Department of Pediatrics, Shaare Zedek Medical Center, Jerusalem, Israel; 3 Microbiology @ Immunology laboratories, Shaare Zedek Medical Center, Jerusalem, Israel; Medical Faculty, Otto-von-Guericke University Magdeburg, Medical Faculty, GERMANY

## Abstract

**Background:**

Congenital Cytomegalovirus (CMV) is a very common intrauterine infection which can cause severe mental and hearing impairments. Notably, only 40% of primarily infected women transmit CMV to the fetus. CMV-specific T-cell response has a role in CMV disease but individual immune heterogeneity precludes reliable correlation between measurable T-cells response and intrauterine transmission.

**Study Aim:**

To establish a correlation between maternal T-cells response and fetal CMV transmission using an individual normalized immune response.

**Methods:**

We analyzed IFN-γ secretion upon whole blood stimulation from primary CMV-infected pregnant women, with either CMV-peptides or PHA-mitogen.

**Results:**

We established a new normalization method of individual IFN-γ response to CMV by defining the ratio between specific-CMV response and non-specific mitogen response (defined as IFN-γ relative response, RR), aiming to overcome high person-to-person immune variability. We found a unique subpopulation of women with low IFN-γ RR strongly correlated with absence of transmission. IFN-γ RR lower than 1.8% (threshold determined by ROC analysis) reduces the pre-test probability of transmission from 40% to 8%, revealing an unexpected link between low IFN-γ RR and non-transmission.

**Conclusion:**

In pregnant women with primary CMV infection, low IFN-γ RR is associated with low risk of transmission.

## Introduction

Cytomegalovirus (CMV) is the most common cause of congenital infection in the developed world, affecting 0.5–2% of all live births in the United States and Europe [1–4]. Fetal CMV infection can cause a variety of long-term disabilities including mental, hearing and visual impairments [5–7]. Severe disabilities caused by congenital CMV infection threaten more children than several well-known childhood maladies such as Down's syndrome or fetal alcohol syndrome [4, 8].

Intrauterine CMV transmission occurs mainly during primary maternal infection, with a maternal-fetal transmission rate of about 40% [8, 9]. The mechanisms dictating CMV intrauterine transmission are unknown. However, transmission is thought to be dependent on multiple factors, including maternal and fetal immune systems, placental factors, maternal viral load and viral strain [9–13].

A large number of studies have demonstrated the essential role of T-cell immunity in the control of CMV infection [12]. It was shown that women with primary CMV infection transmitting the virus to the fetus usually display a delayed T-cell lymphoproliferative response (LPR) to CMV, as compared with non-transmitting women [14–17]. In addition, it has also been reported that circulating CMV-specific effector memory T cells (TEM) may revert to the CD45RA+ phenotype, which is associated with control of CMV viremia and mother-to-fetus transmission[18]. Importantly, individual immune response heterogeneity precludes predicting fetal CMV transmission.

In the current study, we aimed to overcome the personal heterogeneity and find a reliable prediction marker for CMV transmission. We established a novel normalizing value that represents the individual IFN-γ relative response (IFN-γ RR) to CMV peptides. Using this value, we were able to define a subpopulation of women with low transmission rate, characterized by low IFN-γ RR. Pregnant women with primary CMV infection and IFN-γ RR <1.8% (threshold determined by ROC analysis) had a reduced probability of transmission from 40% (pre-test) to 8% [95%CI:1.5%, 30%] (post-test). Our results suggest that low IFN-γ RR reflect an immune state associated with low transmission rate.

## Material and Methods

### Sample collection

Blood samples were sequentially collected from 76 pregnant women diagnosed with primary CMV infection. One woman was excluded from analysis due to spontaneous abortion, and the analysis was performed on 75 women. Primary CMV infection was determined by CMV-specific IgG seroconversion, or the presence of low avidity IgG antibodies or CMV- specific IgM with no previous IgG antibodies. In five random subjects, a repeated blood sample was collected 5–8 weeks following the first sample.

This study was approved by the local ethics committee of Shaare-Zedek Medical Center, written informed consent was obtained from each participant. The study was performed according to the Good Clinical Practice (GCP) guidelines.

### Timing of primary CMV infection and intrauterine transmission

The timing of the primary infection was determined by the time point of seroconversion and/or analysis of the increment of IgG avidity and/or by clinical symptoms [19]. Intrauterine CMV transmission was diagnosed by detection of viral DNA by real-time PCR, either in amniotic fluid or in the newborn’s urine.

### CMV-specific T cell activation

T-cell CMV-specific immunity was assessed by the Quantiferon test (Cellestis, Carnegie, Australia). The collected blood was stimulated with either 22 CMV peptides or with a mitogen (phytohemagglutinin, PHA) for 24 hours at 37°C. In 19 women, blood was collected into additional tube contain *Mycobacterium tuberculosis* (TB) antigens. The T- cell response was determined by measuring secretion of IFN-γ, TNF-α, IL-10 and IL-6 in the supernatant (diluted 1:4).

### IL-6, IL-10, TNF-α and IFN-γ secretion

The concentrations of IL-10, IL-6, TNF-α, and IFN-γ in these samples were determined by the Cytometric Bead Array (CBA) Human Th1/Th2 Cytokine Kit II (BD Biosciences, CA, USA). Briefly, bead populations with distinct fluorescence, coated with capture antibodies specific for IL-6, IL-10, TNF-α, and IFN-γ proteins were pooled together. These beads were mixed with the phycoerythrin-conjugated detection antibodies and were incubated with recombinant standards or test samples. Samples were then analyzed on a FACSCanto^TM^ II flow cytometer (BD Biosciences, CA, USA).

### Peripheral blood mononuclear cells (PBMCs) separation

PBMCs were separated from whole blood with a density gradient tube (Uni-Sep, Novamed) as follows: 10 to 15mL EDTA treated blood were added to the density gradient tube, followed by centrifugation at1000g for 30 minutes. PBMC layer was then carefully removed. Cells were washed twice with PBS and 200µl of RNA save (Biological Industries, Beit Haemek, Israel) were added. Samples were kept overnight at 4°C and stored at –80°C.

### Expression analysis of PD-1

Total RNA was extracted from the separated PBMC using TRI-reagent (Sigma, St. Louis, MO, USA) as described by the manufacturer. For each woman, 150ng of RNA was reverse transcribed by qPCRBIO cDNA synthesis kit (PCRBiosystems, London, United Kingdom). Real time PCR of PD-1 was performed by TaqMan® Gene Expression Assays (Lifetechnology, MA, USA). The β-Glucuronidase (GUSB) gene was used as a control for ΔCt analysis. All qRT-PCR assays included Non-Template Control (NTC).

### Statistical analysis

Statistical analysis was performed using SPSS software V.17 (SPSS, Chicago, Illinois, USA). Continuous variables were compared with *t*-test for normally distributed variables and the Mann–Whitney U test otherwise. Categorical variables were compared with χ2 test.

A logistic regression model was constructed with transmission as the dependent variable and IFN-γ RR as the independent variable, controlling for potential confounding by age, week of infection, gestational week at blood collection, presence of viremia and avidity index.

We examined the diagnostic performance of IFN-γ RR using a receiver operating characteristic (ROC) curve. Optimal IFN-γ RR threshold was chosen at a level of 1.8% in order to yield maximal sensitivity (even at the cost of relatively low specificity) and obtain a high negative predictive value, which defines the pregnant women with low likelihood of transmission. Using this threshold, we determined sensitivity, specificity, positive predictive value and negative predictive value for transmission. All reported *p*-values are two-sided and considered significant at *p*<0.05.

## Results

**Fig 1:**
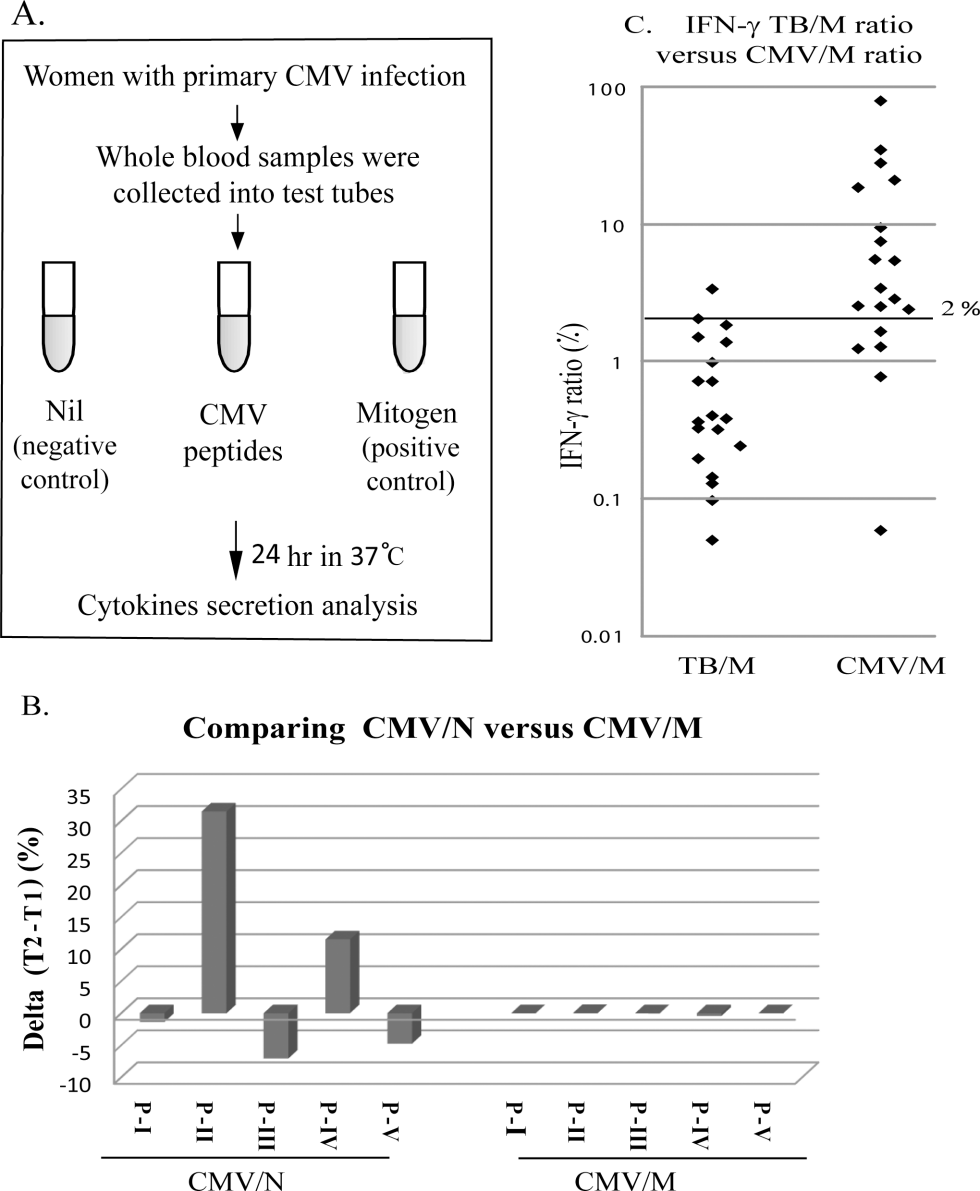
Normalization method of individual IFN-γ specific response. A. Schematic representation of the *in-vitro* stimulation assay. Blood samples of 76 pregnant women with primary CMV were stimulated with either CMV peptides or PHA, for 24 hours at 37°C. The amounts of IFN-γ, TNF-α, IL-10 and IL-6 in the supernatant were determined by CBA analysis. B. Comparing normalizations of IFN-γ induced after CMV stimulation to Nil or Mitogen tubes. We compared the ratios between the levels of IFN-γ induced by CMV peptides divided by that of 'nil' (CMV/N) or 'Mitogen' (CMV/M). Blood samples of five patients (P-I to P-V) were taken in two different time points, T1 and T2 (intervals of 5–8 weeks). Delta determined as the ratio at T1 subtracted from the ratio at T2. C. Comparing IFN-γ TB/M to IFN-g CMV/M ratio. Blood samples of 19 women were collected into Nil, CMV and mitogen tubes, and an additional tube containing Mycobacterium tuberculosis (TB) antigens. We compared the IFN-γ induced by CMV peptides to the IFN-γ induced by TB peptides both normalized to the amount of IFN-γ in the Mitogen tube.

Blood samples were sequentially collected from 75 pregnant women diagnosed with primary CMV infection. CMV transmission occurred in 30 (40%) out of 75 pregnancies. The blood samples were incubated overnight in the presence of: (1) no peptide, (2) CMV synthetic peptides (3) PHA mitogen. Levels of IFN-γ, TNF-α, IL-6 and IL-10 were analyzed in the supernatants to examine the T-cell response (Fig 1A).

We first tested IFN-γ secretion, as a hallmark of immune response against viral infection; in particular, IFN-γ is known to be produced upon lymphocyte activation by CMV peptides [20]. In 66/75 (88%) of the tested women, incubation of whole blood with CMV peptides induced higher secretion of IFN-γ than the IFN-γ level measured in the no peptides-containing tube.

In most previous studies, the immune response to CMV peptides was normalized to the non-activated tube, which subtracts the background. However, individual immune responsiveness is affected by multiple factors including genetics, random infections and microbiota / virome, environmental factors and time post-infection of a given individual. In a survey for a simplified integrated measurement of these parameters we inquired for an *'intrinsic individual normalization'*.

Five pregnant women with primary CMV infection were tested twice within a few weeks interval. We compared two normalization methods: 1. The ratio between the levels of the IFN-γ secreted by cells induced by CMV peptides (specific CMV response) divided by that of the non-activated cells tube ('nil') considered here as background (CMV/N). 2. The ratio between the levels of the IFN-γ secreted by cells induced by CMV peptides to that induced by PHA mitogen, representing the ratio of specific to non-specific stimulation (CMV/M). The rationale of this normalization method was that the specific response to CMV should be compared to the general potential immune response of the same individual. We found that the CMV/M ratio was much less variable within a period of several weeks than the values of CMV/N (Fig 1B). Thereby, we chose the CMV/M ratio, defined here as IFN-γ 'relative response' (RR), to evaluate individual specific response to CMV. To further validate this new normalization method, blood specimens from 19 women were collected into an additional tube containing peptides of *Mycobacterium tuberculosis* (TB). In this non-TB infected cohort, 89% (17/19) of all tested women had TB/M response lower than 2%. In contrast, the CMV/M IFN-γ ratio of the majority of the women (14/19, 73.6%) was higher than 2% (Fig 1C). This result suggests that the IFN-γ RR indeed reflects specific response of the immune system.

Before evaluating the IFN-γ RR of transmitter (T) versus non-transmitter (NT) ratio, we compared general and immunological parameters of these two groups. Both groups were similar in their mean age and the time interval between onset of infection and blood collection (Table 1). Complete blood cell counts and anti-CMV antibodies level (IgG and IgM) were similar as well. Mean IgG avidity index was similar in both groups, in the majority of women it was <20% (T—28/30, 90%, NT—34/45, 75%), suggesting recent CMV infection. Consistent with current knowledge [21–23], mean gestational age at onset of infection was significantly higher in transmitters. Thus, transmission rate during the first trimester (12/43, 27.9%) was lower than that of the second trimester (17/30, 56.6%). In line with previous studies [16, 22] within the sub-group of viremic women, the average CMV copy number was higher in the transmitters compared to the non-transmitters, but this result was not statistically significant (Table 1).

**Table 1:** Clinical and laboratory characteristics of CMV-infected pregnant women- transmitters and non-transmitters.

	Transmitters Mean± SD (n = 30)	Non-transmitters Mean± SD (n = 45)	*P*-value
**Age**	30.3± 4 (22–37)	29± 4.8 (21–40)	NS
**Gestational age at onset of CMV infection (weeks)**	14.5± 7 (1–28)	10.1± 7 (1–28)	0.01
**Gestational age at blood collection (weeks)**	21.4± 6.3 (10–34)	16.8± 6.6 (8–32)	0.003
**Time interval between the infection onset and blood collection (weeks)**	6.8± 3.7 (3–20)	6.6±2.3 (2–12.5)	NS
**IgG avidity index**	12%± 10% (2–43%)	14%±8% (0–41%)	NS
**Women with no CMV copies**	33.3% (10/30)	47.7% (21/44)	-
**CMV copies in viremic women (per ml blood)**	10*10^3^± 38*10^3^ (202–173*10^3^)	3*10^3^± 10*10^3^ (230–48*10^3^)	NS

NS = not significant

**Fig 2:**
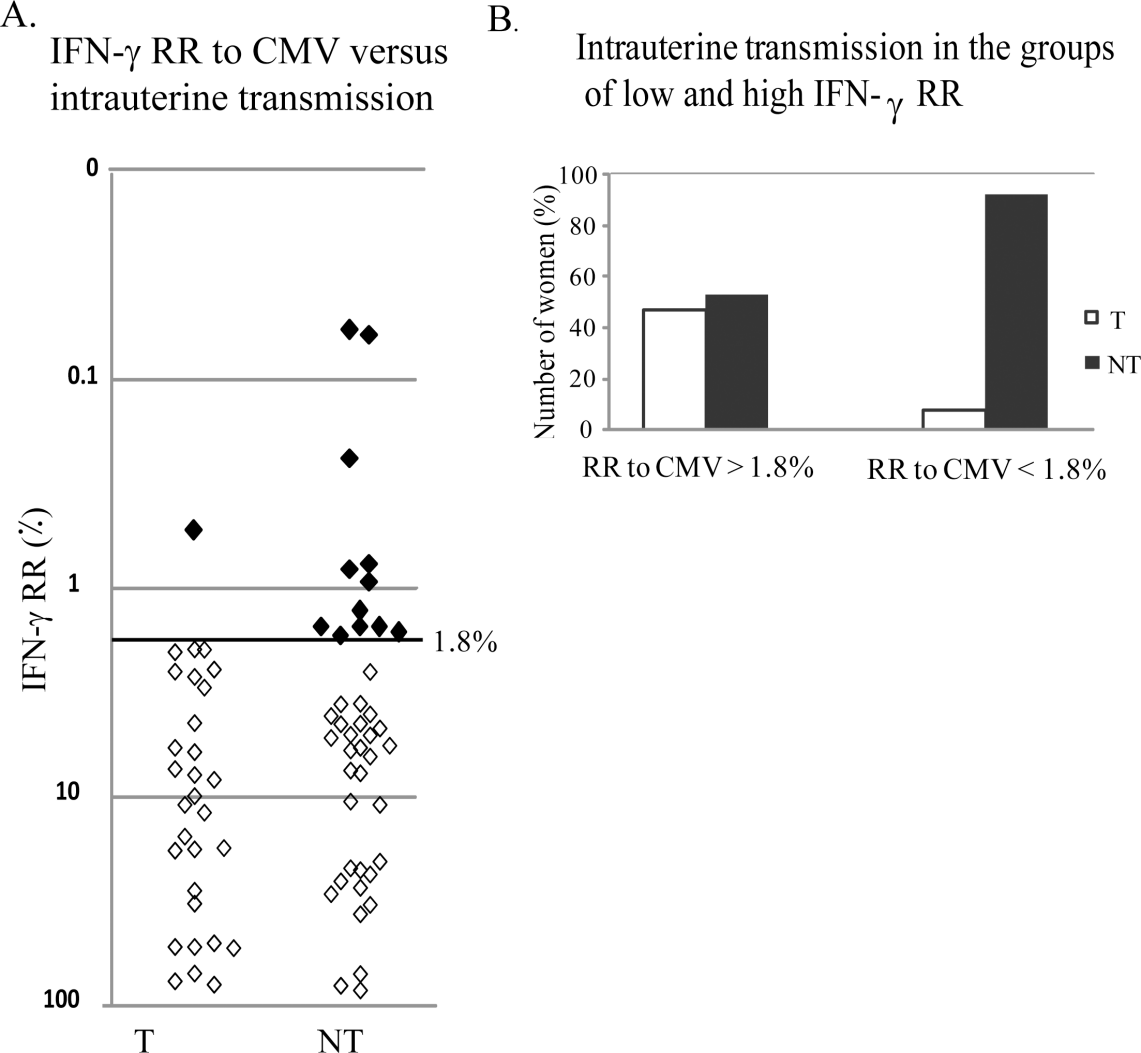
IFN-γ relative response (CMV/M) to CMV and maternal-fetal transmission. A. IFN-γ RR to CMV versus transmission in a continuous distribution, show the individual values. Empty diamonds = high RR, Filled diamonds = low RR. B. Dichotomic distribution of transmitters and non transmitters among women with low (<1.8%) and high (>1.8%) IFN-γ RR, suggesting potential clinical application. IFN-γ RR>1.8%: 29/30 transmitters and 33/45 non-transmitters. IFN-γ RR<1.8%: 1/30 transmitters and 12/45 non-transmitters. RR = relative response, T = transmitters, NT = non transmitters.

We used the IFN-γ RR values for comparing the specific response to CMV of transmitters and non-transmitters women. Unlike CMV/N values that display large overlap between transmitters and non-transmitters (S1 Fig), the distribution of IFN-γ RR values seems different in these two groups. As expected from women who were recently infected by CMV, the IFN-γ RR to CMV of most of the women was high. However, there was a unique group of women with low IFN-γ RR (Fig 2A).

To further describe this sub-population of women displaying low level response to CMV stimulation (i.e. low IFN-γ RR ratio), ROC curve was constructed and threshold was determined at 1.8%. Optimal IFN-γ RR threshold was chosen to yield maximal sensitivity (even at the cost of relatively low specificity) and in order to obtain a high negative predictive value. Using the 1.8% threshold for IFN-γ RR, we found 13 of the 75 women (17.3%) had low IFN-γ RR <1.8% (Fig 2A, full squares and 2B). Interestingly, 12 of these women did not transmit the virus to the fetus. Thus, low IFN-γ RR appeared almost solely among the non transmitters (Fig 2A and 2B).

**Fig 3:**
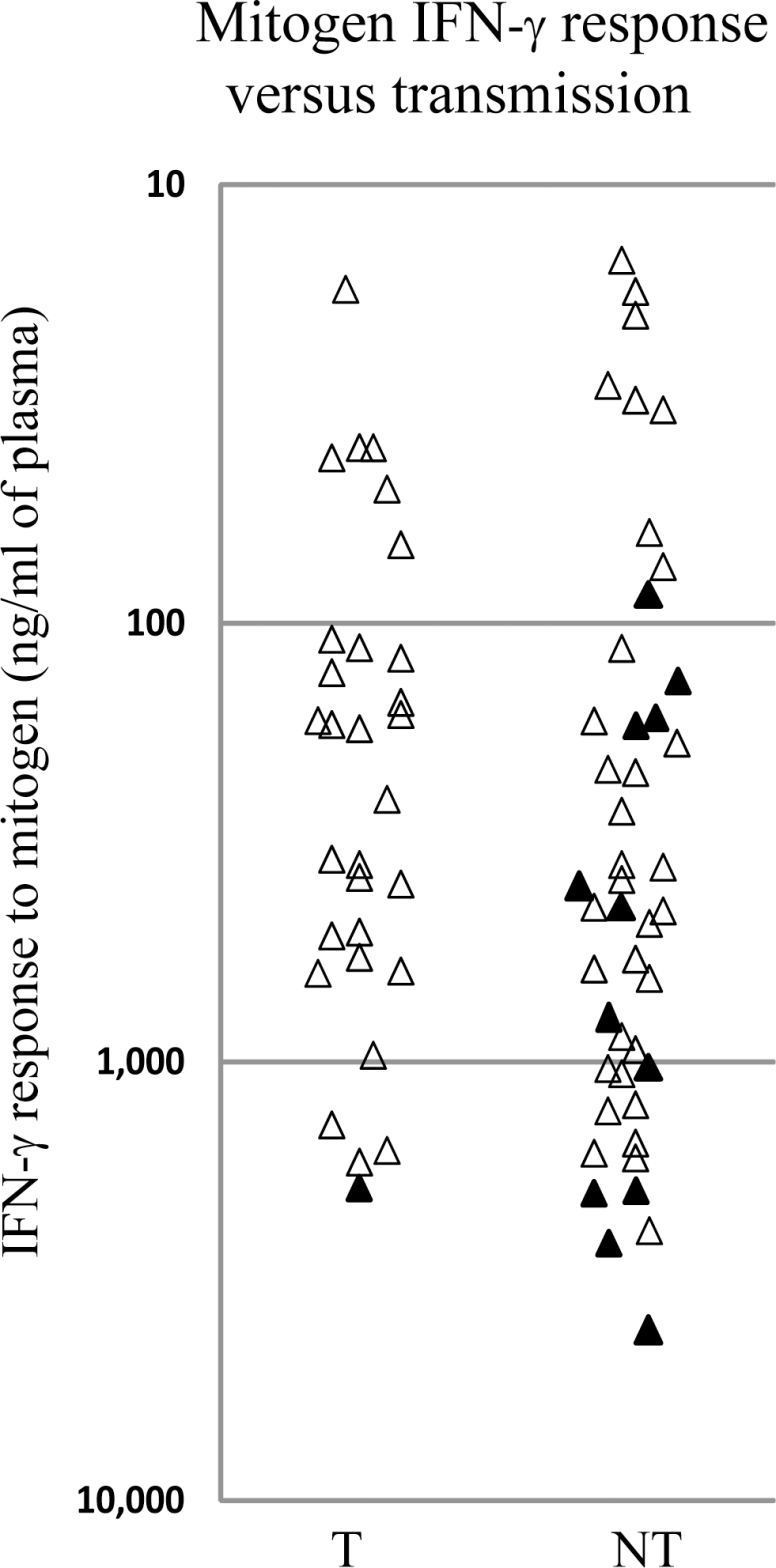
Low IFN-γ RR in non transmitters does not stem primarily from high IFN-γ level caused by mitogen activation. Comparison of IFN-γ levels after mitogen activation in transmitters and non transmitters. Women with IFN-γ RR <1.8% are marked with filled triangles. T = transmitters, NT = non transmitters.

We next tested whether the low IFN-γ RR is due to a high IFN-γ level after mitogen stimulation, leading to high denominator in the CMV/M ratio. IFN-γ values were compared after mitogen activation of transmitters and non-transmitters. As shown in Fig 3, IFN-γ mitogen response values of women who had IFN-γ RR <1.8% (marked with filled triangles) were distributed along the scale. Therefore, it is clear that low IFN-γ RR does not stem primarily from high IFN-γ level caused by mitogen activation.

Next we have examined the association of IFN-γ RR with CMV transmission using a logistic regression model, adjusting for age, gestational age at CMV infection, gestational age at blood collection, avidity index and viremia. Overall, IFN-γ RR>1.8% is positively associated with CMV transmission (OR = 12.9, 95% CI: 1.3,120, p = 0.02). Analysis of the subgroup of women with low avidity and the subgroup with viremia did not essentially change this association.

When additional cytokines of women with RR<1.8% were tested, no correlation between low IFN-γ RR and low TNF-α, IL-10 and IL-6 RRs to CMV challenge was detected. These results suggest that the low IFN-γ RR does not stem from an overall low immune status, but rather represents a more specific phenomenon.

**Fig 4:**
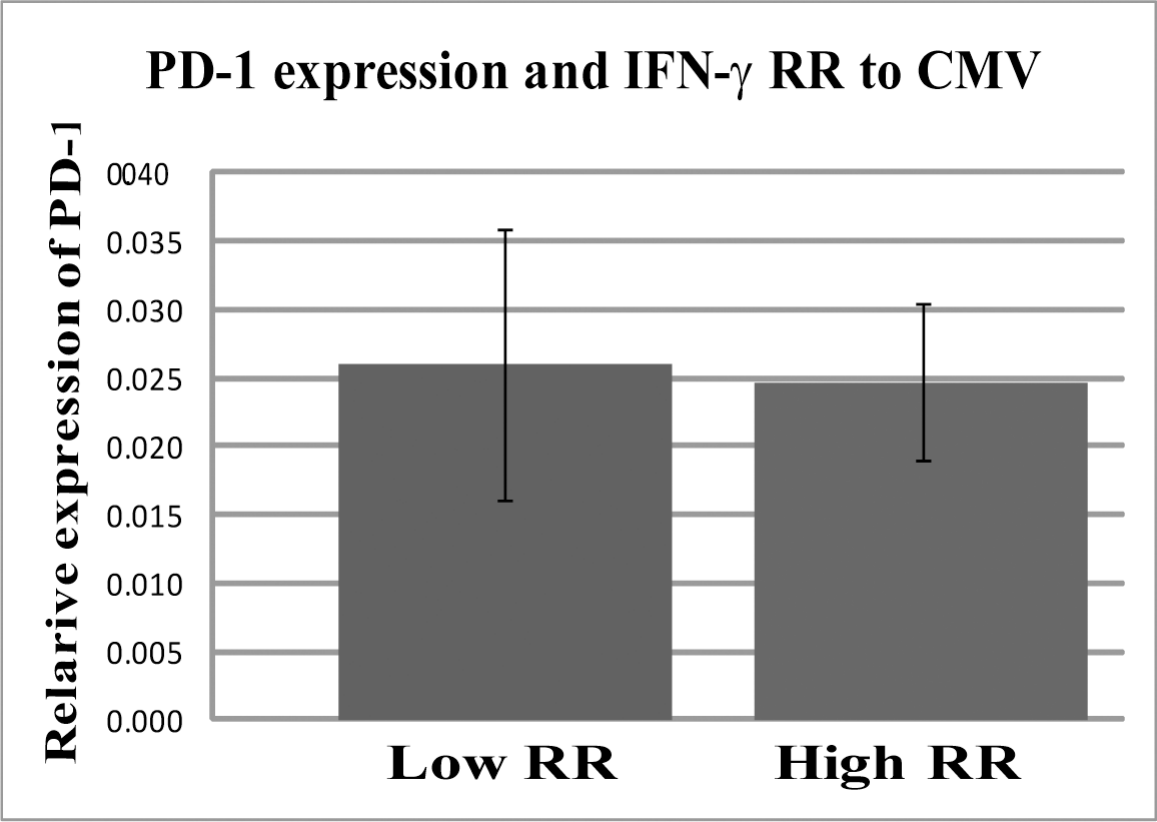
PD-1 expression in PBMC’s of women with high and low IFN-γ relative response is similar. The expression of PD-1 in PBMC was performed by TaqMan Real time PCR of PD-1 and GUSB (see methods section). ∆Ct was calculated (average ∆Ct of PD-1minus the average ∆Ct of GUSB). Relative expression (2^(-∆Ct)±SE) values of women with high and low IFN-γ RR were compared by *t*-test.

One possible explanation for low levels of IFN-γ RR among non-transmitters is the exhaustion of maternal T-cell response which is known to represent a particular state of T-cell dysfunction, characterized by high levels of PD-1 [24, 25]. To test this possibility we performed real-time RT-PCR analysis of PD-1 mRNA levels in the PBMCs from the patients. No significant differences were found between transmitters and non-transmitters as well as between women with high and low levels of IFN-γ RR (Fig 4).

**Fig 5:**
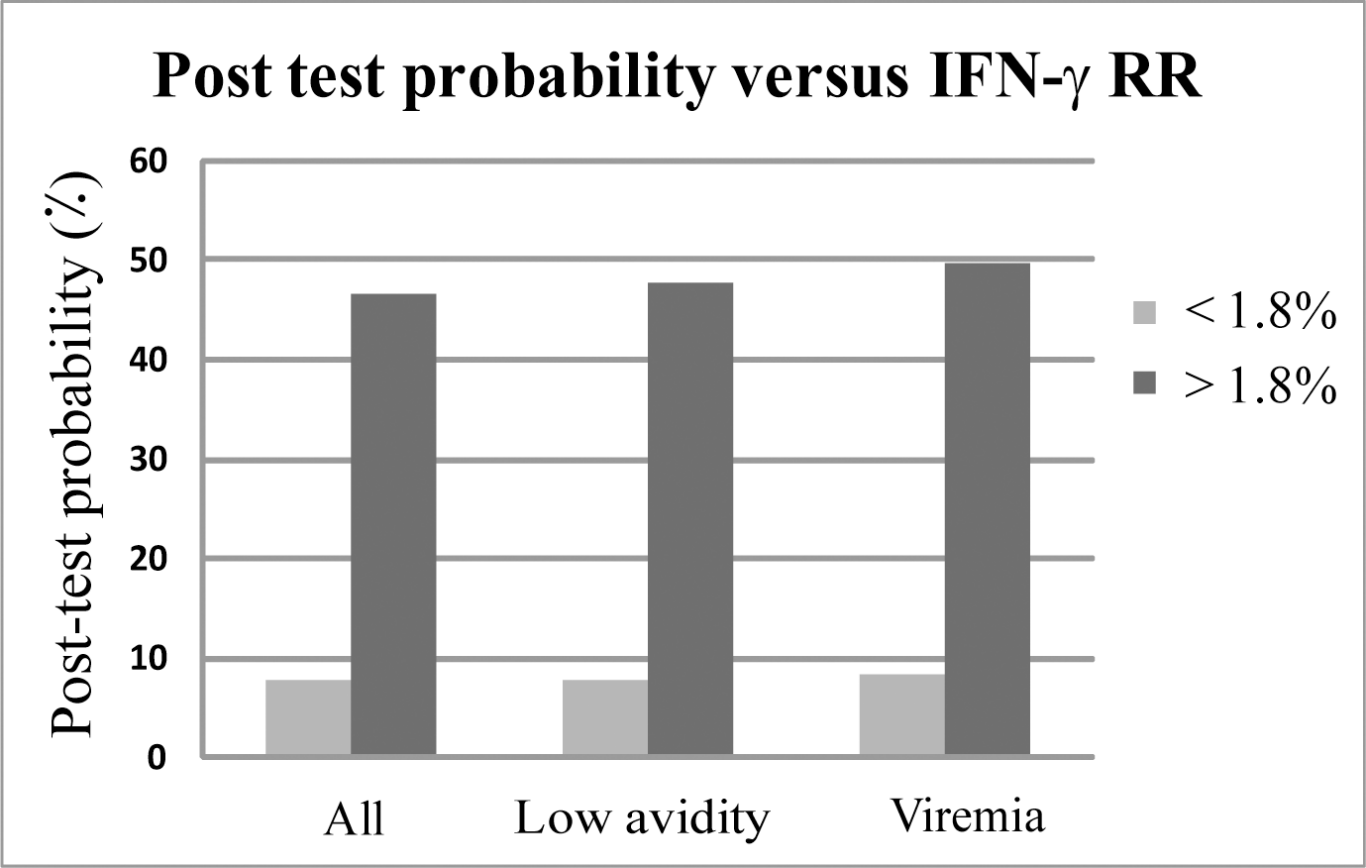
Post-test probability of women with low IFN-γ relative response to CMV. The post-test probability of women with IFN-g RR<1.8% reduced from 40% to 7%-8% in all tested women (n = 75), for women with low IgG avidity (n = 62) and for viremic women (n = 37).

Applying our data to clinical practice, IFN-γ RR <1.8% predicts non-transmission with a sensitivity of 96% (95% CI: 83.3, 99.4) and specificity of 26% (95%CI: 16, 41) resulting likelihood ratio of 0.12 (95% CI: 0.02, 0.9). Given these values, the pre-test probability of CMV transmission of women with RR<1.8% was reduced from 40% to a post-test probability of 8% (95%CI: 0.01, 30) (Fig 5). Thus, the diagnostic yield of the test allows to reduce fivefold the probability of women with primary CMV infection and RR<1.8% to transmit the virus.

## Discussion

This study establishes a new normalization strategy for testing the IFN-γ individual response to CMV peptides in pregnant women infected with primary CMV. Our strategy aims to overcome the high person-to-person immune variability by defining the ratio between specific-CMV response and non-specific mitogen response (termed IFN-γ RR). IFN-γ RR indeed reflects specific activation of the immune system against CMV infection by comparing the response to CMV peptides and TB peptides. Using this new criterion, we revealed a unique subpopulation of women with low IFN-γ RR to CMV (<1.8%) associated with a low likelihood of transmission. Applying these data to clinical practice, the pre-test probability of CMV transmission of women with RR<1.8% was reduced from 40% to 8%.

This work presents a new use of the PHA mitogen response value. In this study, the magnitude of the *in-vitro* response to the mitogen was considered as a quantitative value of the tested individual immune response, rather than just a qualitative -positive control for the *in-vitro* immune response. PHA was used to ensure that the mitogen response is not changing due to the CMV infection, as it has been previously shown that unlike other mitogens, the mononuclear cells response to PHA is similar in CMV patients and healthy donors [26].

The intriguing question posed by our data is how the low immune response (reflected by low IFN-γ RR), can efficiently prevent intrauterine transmission. We addressed here the possibility that low IFN-γ RR stems from T-cells exhaustion. However, we found no significant alterations in PD-1 mRNA levels between women with high or low IFN-γ RR.

These results suggest that low IFN-γ RR does not stem primarily from exhaustion. However, evaluating levels of surface PD-1 expression on a variety of T-cell subpopulations might help to address the possibility of specific exhaustion of a particular T-cells pool. For example, it has been shown that PD-1 expression was increased on CD28+ CMV-specific CD4+ T cells of pregnant women with CMV [27].

Another possible mechanism explaining the reverse correlation we observed could be related to the differences in the efficiency of immune response in viral clearance. Our data suggests that women with low IFN-γ RR provide a very efficient response to the virus infection. This can be due to prompt response after infection, the response intensity or other unknown parameters.

Comparison of the current study results with previous studies, especially the one that has shown lower and delayed LPR to CMV in transmitters women [16], is limited due to technical differences. Two major differences should be considered: first, in our work, T-cell response was tested in whole blood, while in Revello's study the response was tested in isolated PBMCs. Second, we activated the cells for 24 hours while the activation in the Revello's study was for 6 days with CMV and 3 days with PHA. These differences might affect the readout of the cytokine pattern obtained.

We show that IFN-γ RR <1.8% predicts non-transmission with a high sensitivity (96.5%) and high negative predicting value (92%). In our cohort, low IFN-γ RR was associated with a substantial reduction in the probability of CMV transmission (from 40% to 8%). The association of IFN-γ RR with transmission remained unchanged in the sub-groups of women with viremia and low avidity, which further validates our finding. Since our data show that the IFN-γ RR of considerable number of women are centered next to the determined threshold, further research with larger cohort will be needed to substantiate the determined threshold and its potential clinical utilization.

## Conclusions

This study suggests a new way of looking at an old challenge; searching for a correlation between the *in-vitro* IFN-γ response to CMV peptides and intrauterine transmission. We propose a novel normalization method testing the individual response to CMV, and defined a sub-group with a very low likelihood of transmission characterized by low IFN-γ RR to CMV (as measured *in-vitro*). IFN-γ RR might be one of many other factors which together determine intrauterine transmission of CMV following primary infection. In a broader perspective, the approach of simple individual immune normalization introduced here could possibly be applied to other human infections.

## Supporting Information

S1 Fig CMV/N values display large overlap between transmitters and non-transmitters. A continuous distribution of the ratios between IFN-γ induced by CMV peptides divided by that of 'nil'-empty tube (CMV/N) versus intrauterine transmission of CMV. T = transmitters, NT = non transmitters.
(TIF)
