# Supplementary figures and images for: Low Interferon Relative-Response to Cytomegalovirus Is Associated with Low Likelihood of Intrauterine Transmission of the Virus
Source: PLoS One. 2016 Feb 16;11(2):e0147883. doi: 10.1371/journal.pone.0147883 (PMC4755570; doi:10.1371/journal.pone.0147883)

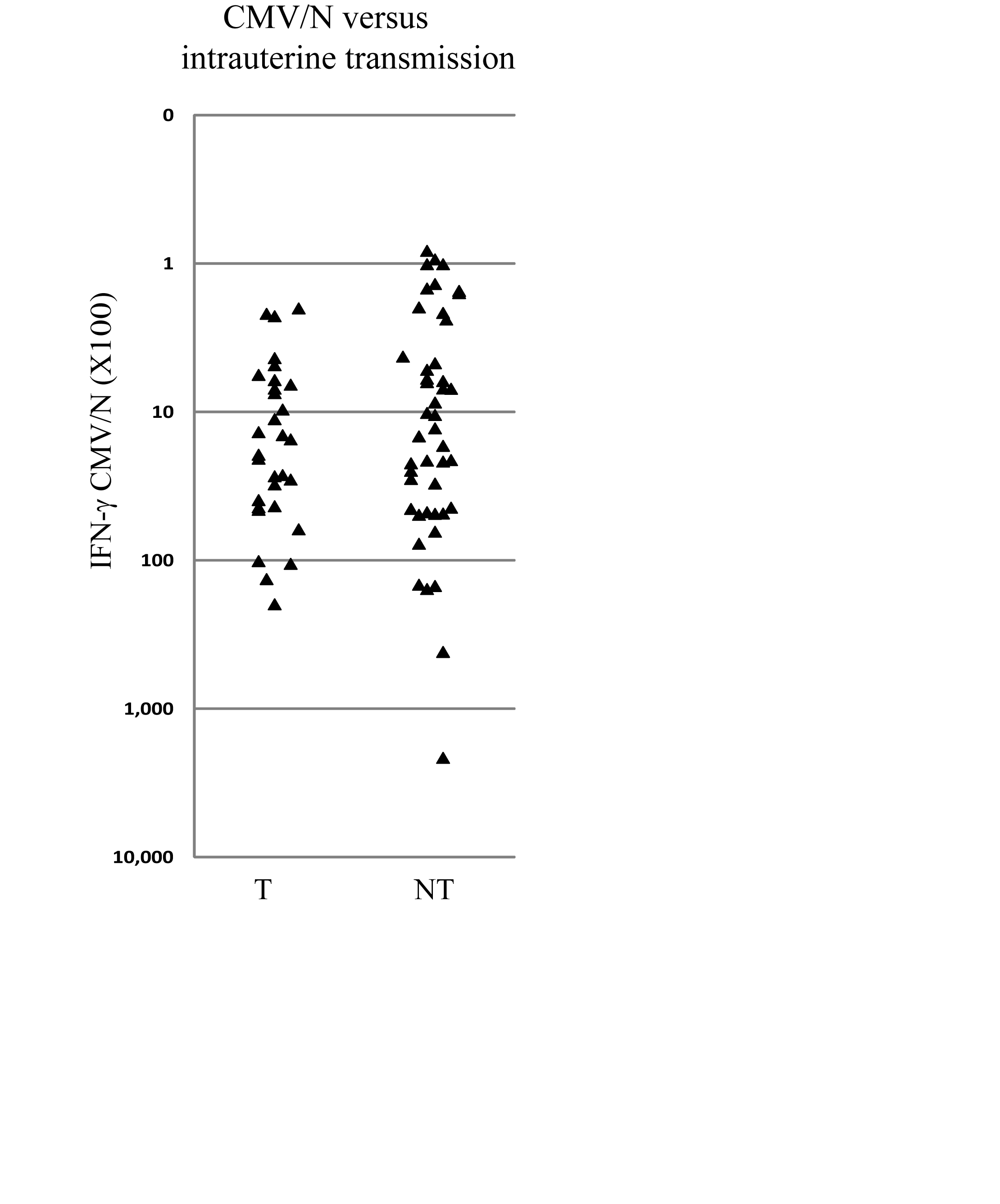

Supplement: S1 Fig — A continuous distribution of the ratios between IFN-γ induced by CMV peptides divided by that of 'nil'-empty tube (CMV/N) versus intrauterine transmission of CMV. T = transmitters, NT = non transmitters. (TIF) [file pone.0147883.s001.tif]
